# Experimental Tests of Priority Effects and Light Availability on Relative Performance of *Myriophyllum spicatum* and *Elodea nuttallii* Propagules in Artificial Stream Channels

**DOI:** 10.1371/journal.pone.0120248

**Published:** 2015-03-19

**Authors:** Emily P. Zefferman

**Affiliations:** Department of Plant Sciences and Graduate Group in Ecology, University of California Davis, Davis, California, United States of America; Shandong University, CHINA

## Abstract

Submersed macrophytes have important ecological functions in many streams, but fostering growth of beneficial native species while suppressing weedy invasives may be challenging. Two approaches commonly used in management of terrestrial plant communities may be useful in this context: (1) altering resource availability and (2) establishing desirable species before weeds can invade (priority effects). However, these approaches are rarely used in aquatic systems, despite widespread need for sustainable solutions to aquatic weed problems. In artificial stream channels in California, USA, I conducted experiments with asexual propagules of non-native invasive *Myriophyllum spicatum* (Eurasian watermilfoil) and native *Elodea nuttallii* (western waterweed) to address the questions: (1) How does light availability affect relative performance of the two species?; (2) Does planting the native earlier than the invasive decrease survival or growth rate of the invasive?; and (3) Do light level and priority effects interact? The relative performance between *E*. *nuttallii* and *M*. *spicatum* had an interesting and unexpected pattern: *M*. *spicatum* had higher growth rates than *E*. *nuttallii* in the zero and medium shade levels, but had similar performance in the low and high shade levels. This pattern is most likely the result of *E*. *nutallii’s* sensitivity to both very low and very high light, and *M*. *spicatum’s* sensitivity to very low light only. Native priority did not significantly affect growth rate or survival of *M*. *spicatum*, possibly because of unexpectedly poor growth of the *E*. *nuttallii* planted early. This study suggests that altering light levels could be effective in reducing growth of an invasive macrophyte, and for changing the competitive balance between a native and a non-native species in the establishment phase. Further investigations into the use of priority effects and resource alteration for submersed macrophyte management are warranted, given their mixed results in other (limited) studies.

## Introduction

The role of resource availability in determining relative performance of plant species is often examined in the context of competition between native and non-native invasive species (hereafter, ‘invasives’). Higher resource levels are commonly thought to favor invasive over native species, as plant traits that confer invasiveness (e.g., high growth rates and fecundity) are often associated with higher resource use [1,2]. Many studies do show a competitive advantage of invasive species over natives in high resource conditions and vice-versa [3]. However, invasives in certain ecosystems can be more efficient at using scarce resources than natives, thus outperforming natives in *lower* resource conditions [4–6]. Research into the competitive outcomes between natives and invasives across resource levels has focused largely on terrestrial plant communities, with relatively little attention to aquatic plant (submersed macrophyte) communities, particularly in flowing systems.

Light is an important limiting resource in freshwater systems [7] and can be manipulated through management of canopy-forming riparian vegetation. Reducing light by increasing canopy shading has been recommended for controlling growth of submersed macrophytes generally [8–10]. However, few, if any, studies specifically address how reductions in light may affect *relative* performance of invasive over native macrophytes when grown in a competitive environment.

Native submersed macrophytes play an important and beneficial role in many streams, providing food and habitat for aquatic organisms and modifying the physical and chemical environment [11,12]. Invasive macrophytes, however, can have large negative economic and ecological impacts in natural and human-made waterways [9,13], and have been linked with reduced abundance and/or diversity of native macrophytes [14,15], invertebrates [16,17] and fish [16,18]. Controlling invasive macrophytes through traditional physical or chemical means can pose logistical difficulties in flowing systems due to the potential for dispersal of unintentionally-created vegetative propagules and reduced contact time with herbicides [9]. In addition, these methods often cause collateral damage to other aquatic organisms and generally have only short-term impacts [9]. Therefore, understanding how manipulating resource availability affects the competitive balance between native and invasive macrophytes should be useful to managers of flowing waters who are interested in preserving or restoring native diversity while reducing the impact of invasives.

In addition to resource availability, priority effects—the impacts of species arrival and establishment order on community structure—are known to affect the relative performance of species. The idea that early-arriving species can preempt resources, reducing the success of later arrivers, is a basic tenet of community assembly theory [19]. Priority effects have been studied extensively in terrestrial plant communities, often in the context of restoration and with a goal of giving native species a competitive edge over invasive species [20]. Yet, the role of priority in determining macrophyte community composition and the potential for using priority to achieve management outcomes have only just begun to be addressed in freshwater systems [21,22].

Priority effects may be particularly relevant after stream restoration, as these projects may involve large disturbances (e.g., temporary drying of stream channels or creation of new channels) that ‘reset’ macrophyte communities by killing off resident species or creating new substrate. While active planting of riparian vegetation often takes place after stream restoration, submersed macrophytes are typically expected to recolonize on their own [21]. If actively planting native macrophytes early could effectively reduce the impact of invasive macrophytes, it would be a useful management tool.

Perhaps as interesting as the individual effects of either altering resource availability or giving native macrophytes a temporal priority advantage is how these two potential management techniques could interact. For example, priority effects may be stronger in higher light environments if early-arriving species are able to preempt other resources (e.g., soil nutrients) more quickly. Interactions between priority effects and resource manipulation are rarely studied (but see [23,24]) and to my knowledge, have never been studied in aquatic systems.

To investigate the effects of light level (resource availability), temporal advantage (priority effects), and their potential interaction on the relative performance of native and non-native submersed macrophytes during initial establishment, I conducted a mesocosm experiment in central California, USA, using native *Elodea nuttallii* (western waterweed), and invasive *Myriophyllum spicatum* (Eurasian watermilfoil) as study species. In North America, *M*. *spicatum* is considered one of the most pernicious non-native invaders of fresh waters [25]. *Elodea nuttallii* is a common native in California, and co-occurs with *M*. *spicatum* in local waterways [26]. Both are perennials that reproduce primarily through dispersal of vegetative fragments [27]. Because *M*. *spicatum* has been associated with altered fish and invertebrate assemblages [16,28,29] and declines in native macrophyte richness [30,31], the competitive outcomes between these species could have important effects on aquatic communities. To date, most experiments on submersed macrophytes, including these two species, have been conducted in laboratory conditions with little water flow and under artificial light. Because I wanted my experimental results to be applicable to flowing systems, I created artificial stream channels for these experiments under natural light conditions.

I predicted that (1) decreasing light availability (by increasing shading) would improve performance of the native *E*. *nuttallii* relative to the invasive *M*. *spicatum*; (2) planting *E*. *nuttallii* earlier than *M*. *spicatum* (priority advantage) would reduce survival and growth rates of *M*. *spicatum*; (3) giving *E*. *nuttallii* an establishment priority would have a greater suppressive effect on *M*. *spicatum* in high light treatments than in the high shade treatments (a priority x light availability interaction).

## Methods

### Site description

**Fig 1:**
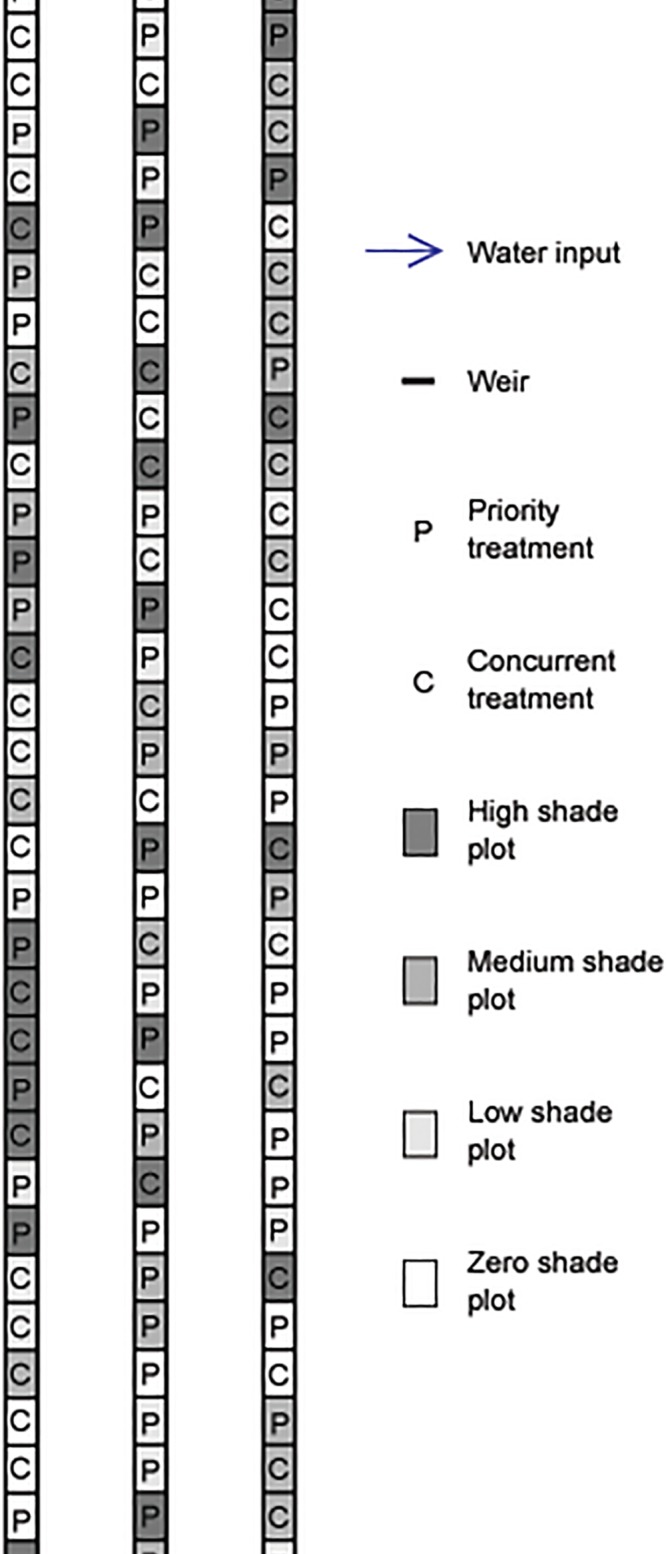
Diagram of experimental layout in artificial channels. Water flowed through the entire channel system with inputs at the upstream end of each of the three channels.

A system of connected artificial stream channels was created for this experiment at the University of California, Davis (UC Davis) Putah Creek Riparian Reserve in the Central Valley of California (38.52833°N, 121.78528°W, elevation 21 m). All of the methods described herein took place within, and were approved by, the Putah Creek Riparian Reserve. Three channels were excavated in a field dominated by grasses and forbs with soil classified as Yolo Silt Loam. Each channel was approximately 60 m long, 1 m wide, 0.6 m deep (water depth range = 0.32–0.48 m). Water from an aquaculture facility was diverted to flow through all three channels, with water inputs at the upstream end of each channel (Fig. 1). The channels were flooded for 17 days prior to initial planting. Water velocity averaged 0.05 m/s throughout the experiment and was slightly higher near the three water inputs and slightly lower at the downstream ends of the channels.

To characterize the nutrient levels in the system, I collected soil samples from five different locations within the channels on 7 September 2012, and took water samples from upstream, middle, and downstream locations in the channel system on 18 September 2012 and again on 23 September 2012. Samples were analyzed by the UC Davis Analytical Laboratory (http://anlab.ucdavis.edu) for ammonium and nitrate (SOP 312 for soil, SOP 847 for water), and phosphorus (SOP 340 for soil, SOP 865 for water). Sediment and water nutrient levels were similar throughout the channel system (Table 1). Typical values for specific conductance, hardness, and pH in the source water for this system are, respectively, 750 μS/cm, 350 mg/L (CaCO_3_), and 8.1 SU (personal communication, Paul Lutes, UC Davis Center for Aquatic Biology and Aquaculture).

**Table 1:** Mean values and ranges of key nutrient levels of water and soil samples in the experimental channels.

Parameter	Mean	Range
***Water***		
Ammonium-N (mg/L)	<0.05*	NA
Nitrate-N (mg/L)	6.24	(6.19–6.33)
Orthophosphate-P (mg/L)	0.07**	(<0.05–0.10)
***Soil***		
Ammonium-N (ppm)	2.95	(2.09–3.78)
Nitrate-N (ppm)	2.18	(1.55–2.61)
Phosphorus (Olsen-P) (ppm)	6.79	(5.05–8.20)

*Ammonium was not detected (mdl = 0.05 mg/L) in any samples.

**Three samples had orthophosphate levels below detection limits (0.05 mg/L)

I deployed HOBO U22 Water Temp Pro v2 temperature loggers (Onset Computer Corporation, Cape Cod, MA) in the middle of each channel from 18–29 September 2012, and recorded temperatures every two hours. Temperatures were similar among channels (within 0.25°C difference on average), and ranged from daily lows around 18.0°C to highs around 20.6°C.

### Experimental Design

Using a completely randomized design, I assigned one of eight treatment combinations—two competition treatments crossed with four shade treatments—to 1.5 m-long plots in the channel system, with 14 replicates per treatment. In both competition treatments, three 5 cm stem fragments of *E*. *nuttallii* and three 5 cm stem fragments of *M*. *spicatum* were planted into each plot by staking each fragment to the sediment surface, but in the ‘priority’ competition treatment *E*. *nuttallii* was planted 35 days before *M*. *spicatum*, while in the ‘concurrent’ competition treatment both species were planted at the same time. In each plot, stem fragments were placed 5 cm apart alternating species in a 2x3 arrangement. The timing, number, and spacing of plants was informed by observed growth of the same two species in a study conducted the previous year [32].

I collected macrophytes locally from Putah Creek (38.52667°N, 121.80361°W) and removed all attached algae, invertebrates, and/or roots before cutting stems into 5 cm fragments. All fragments were apical. Five cm is in the modal range for naturally dispersing vegetative propagules of *E*. *nuttallii* and *M*. *spicatum* found locally in Putah Creek [33]. Ten additional 5 cm fragments of each species were dried at 60°C to determine average initial weights for calculating relative growth rates.

Shade treatments were implemented using black polyethylene shade cloth of three different weights—30% (‘low’), 60% (‘medium’), or 90% (‘high’)—or no shade cloth (‘zero’). Previous work [32]determined that the actual amount of photosynthetically active radiation (PAR) reduced by the 30%, 60%, and 90% shade cloth was on average 40%, 72%, and 94%, respectively. Shade cloth was secured over the plots approximately 0.2 m above the surface of the water after planting. To determine the amount of light reaching the planted macrophytes under each shade treatment, I measured PAR below the water on 14 and 18 August 2012 between 12:00pm and 12:30pm with a LI-COR LI-193 spherical quantum sensor (LI-COR, Lincoln Nebraska, USA).

*Elodea nuttallii* was planted into the priority plots on 13 and 14 July 2012. On 17 and 18 August 2012, *M*. *spicatum* was planted into the priority plots, and all of the concurrent plots were planted with both species. On 29 and 30 September 2012 (43 days after planting *M*. *spicatum* in both treatments), biomass was harvested at the plot level. Some sections of the channel walls caved in during the experiment, covering the macrophytes in some plots. Biomass was only collected from undisturbed plots, reducing the replicates for some treatments to 12 or 13 (Table 2). All above-soil biomass was collected in each harvested plot; the species were separated, placed into paper bags, dried for 48 hrs at 60°C in a drying oven, and weighed.

**Table 2:** Means, standard errors, and number of replicates for each species in each treatment combination.

Shade level	Species	Competition treatment	RGR (g/g/d)	Biomass (mg)	N
Zero	*Elodea nuttallii*	Concurrent	0.0667 ± 0.0055	882.9 ± 374.4	12
		Priority	0.0330 ± 0.0049	920.9 ± 354.6	14
	*Myriophyllum spicatum*	Concurrent	0.0795 ± 0.0021	1945.6 ± 175.5	12
		Priority	0.0831 ± 0.0019	2268.3 ± 200.3	14
Low	*Elodea nuttallii*	Concurrent	0.0767 ± 0.0067	1404.6 ± 462.4	12
		Priority	0.0410 ± 0.0051	1602.3 ± 659.7	12
	*Myriophyllum spicatum*	Concurrent	0.0799 ± 0.0025	2011.5 ± 201.1	12
		Priority	0.0764 ± 0.0026	1749.3 ± 199.1	12
Medium	*Elodea nuttallii*	Concurrent	0.0599 ± 0.0044	496.0 ± 82.0	12
		Priority	0.0450 ± 0.0065	5493.6 ± 3108.2	14
	*Myriophyllum spicatum*	Concurrent	0.0721 ± 0.0020	1404.2 ± 117.1	12
		Priority	0.0696 ± 0.0018	1267.7 ± 95.6	14
High	*Elodea nuttallii*	Concurrent	0.0452 ± 0.0028	240.1 ± 32.7	12
		Priority	0.0267 ± 0.0036	410.1 ± 122.9	13
	*Myriophyllum spicatum*	Concurrent	0.0422 ± 0.0041	416.8 ± 44.4	12
		Priority	0.0399 ± 0.0040	390.1 ± 47.1	13

### Statistical analysis

Although initial fragment lengths were the same for both species in all treatments, the *E*. *nuttallii* fragments were lower in biomass. To account for this initial difference between species, I used relative growth rate (RGR) as the response variable in all of the analyses, calculated as [ln(final dry weight)—ln(initial dry weight)]/#days. In cases where final biomass was zero or smaller than initial biomass, I assigned a RGR of ‘0’. All analyses were completed using the ‘stats’ package in R (R Development Core Team, Vienna, Austria).

To determine whether shade changed the relative performance of *E*. *nuttallii* and *M*. *spicatum* when grown concurrently, I first used analysis of variance (ANOVA), including shade, species, and their interaction as predictors. I used a weighted least squares approach to accommodate unequal variances between the two species. ANOVA was followed by Tukey means comparisons of RGR across shade levels within each species, and pairwise t-tests comparing *E*. *nuttallii* to *M*. *spicatum* RGR in each shade level. I also tested the correlation between *M*. *spicatum* and *E*. *nuttallii* RGR within each shade level, using Pearson’s product moment correlation coefficient.

To determine whether *M*. *spicatum* growth rates were affected by the timing of *E*. *nuttallii planting*, and whether the magnitude of these effects was influenced by shade level, I used ANOVA with shade, competition treatment, and their interaction as predictors and *M*. *spicatum* RGR as the response variable. To comply with ANOVA assumptions of normality of residuals, I Winsorized the dataset by adjusting the RGR values with the two lowest and two highest residuals to the 2^nd^ and 98^th^ percentiles, respectively [34]. (Winsorizing did not ultimately change the conclusions of the analysis.) ANOVA was followed with means comparisons by Tukey’s Honest Significant Different method.

## Results

Mean PAR levels measured underwater below the zero, low, medium, and high shade treatments at mid-day in mid-August were 1922, 1218, 643, and 233 μmol photons m^-2^ s^-1^, respectively. Final biomass and RGR means and standard errors, and number of replications per treatment are shown in Table 2.

### Effect of shade level on competition between *M*. *spicatum* and *E*. *nuttallii*

**Fig 2:**
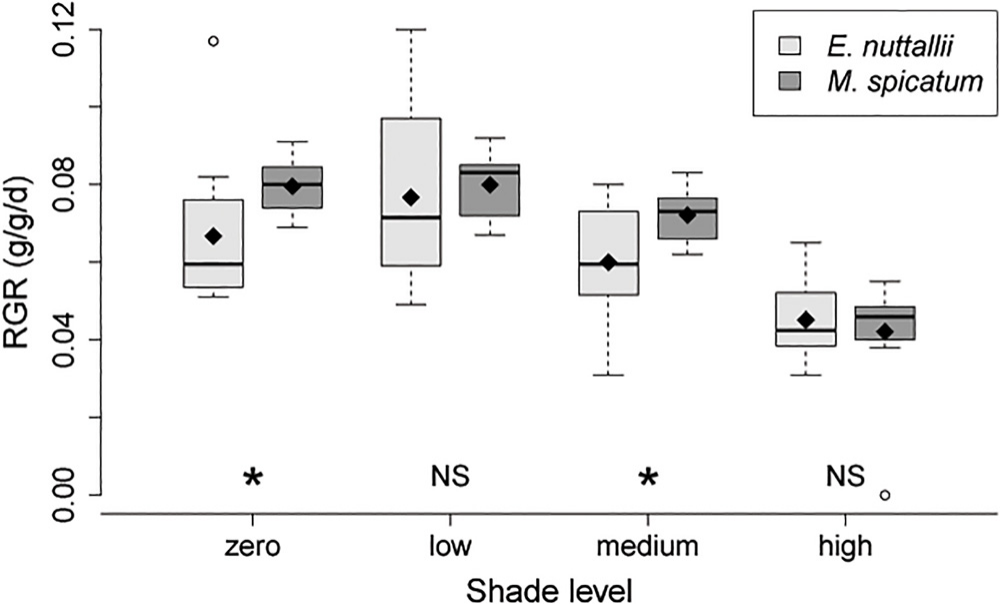
Boxplots comparing RGR of *M*. *spicatum* and *E*. *nuttallii* planted concurrently across shade levels. Box edges mark the 1st and 3^rd^ quartile, and the median is shown with a dark line. Whiskers extend to a maximum of 1.5 x interquartile range outward, and values beyond this are indicated by circles. Diamonds show the mean of each shade level*species combination. Asterisks indicate significant differences (p < 0.05) in pairwise t-tests between species, and “NS” indicates non-significant differences.

Plot level survival was 100% for both species in the concurrent treatment. Overall, *M*. *spicatum* had higher RGR than *E*. *nuttallii* (significant species effect). Shade had a significant effect on RGR, but these differences across shade levels did not differ significantly between the two species (no significant species x shade interaction) (Table 3A, Fig. 2). The mean RGR of *E*. *nuttallii* in the concurrent treatment was highest in the low shade level, intermediate in the zero and medium shade levels, and lowest in the high shade level—41.1% lower than the low shade level (Fig. 2). However, Tukey means comparisons for *E*. *nuttallii* in the concurrent treatment only show a significant difference in RGR in the high shade level compared to the zero (p = 0.022) and low shade levels (p = 0.0004).

**Table 3:** ANOVA statistics.

	Degrees of Freedom	F-statistic	p-value
*A. Effect of shade level and species on RGR in concurrent treatment*			
Shade level	3	45.7	<0.0001
Species	1	4.8	0.03
Shade*Species	3	1.7	0.17
*B. Effect of shade level and competition treatment (priority) on* M. spicatum *RGR*			
Shade level	3	118.8	<0.001
Competition treatment	1	0.6	0.45
Shade*Competition treatment	3	1.0	0.38

Pairwise comparisons of the two species in each shade level found that *M*. *spicatum* RGR was 19% higher than *E*. *nuttallii* RGR in the zero shade treatment (p = 0.029) and 20% higher in the medium shade treatments (p = 0.038). In the low and high shade levels, performance between species was similar (mean difference of *M*. *spicatum—E*. *nuttallii* RGR = 0.003 g/g/d and -0.004 g/g/d, respectively) and was not significantly different (p = 0.58 and p = 0.61, respectively) (Fig. 2).

**Fig 3:**
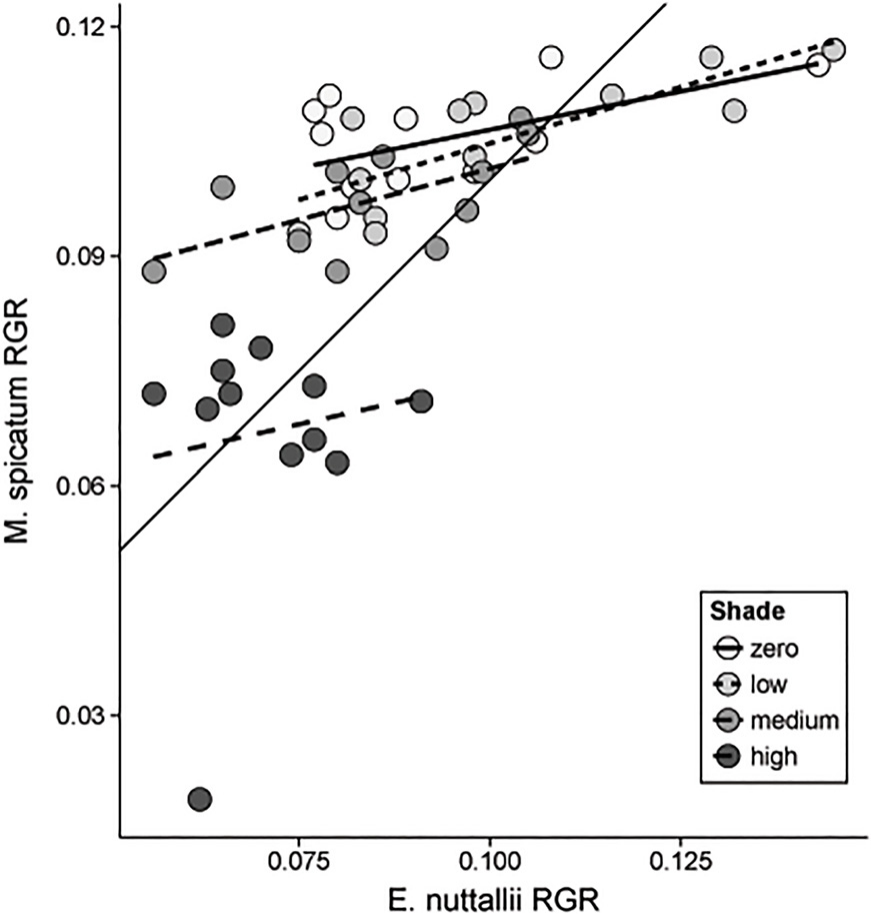
Correlation of *M*. *spicatum* and *E*. *nuttallii* RGR in the concurrent treatment. Thin, solid line is 1:1 line. Most points fall to the upper left of this line, indicating a higher RGR for *M*. *spicatum* over *E*. *nuttallii*. The Pearson correlation coefficient (r) and p-values for each shade level are as follows (DF = 10 for all tests): zero: r = 0.54, p = 0.07; low: r = 0.80, p = 0.002; medium: r = 0.61, p = 0.04; high: r = 0.14, p = 0.68.

*Myriophyllum spicatum* and *E*. *nuttallii* RGR were positively correlated in all shade levels within the concurrent treatment, although the correlations were only significant in the medium and low shade levels (Fig. 3).

### Effect of native priority and shade on M. spicatum

**Fig 4:**
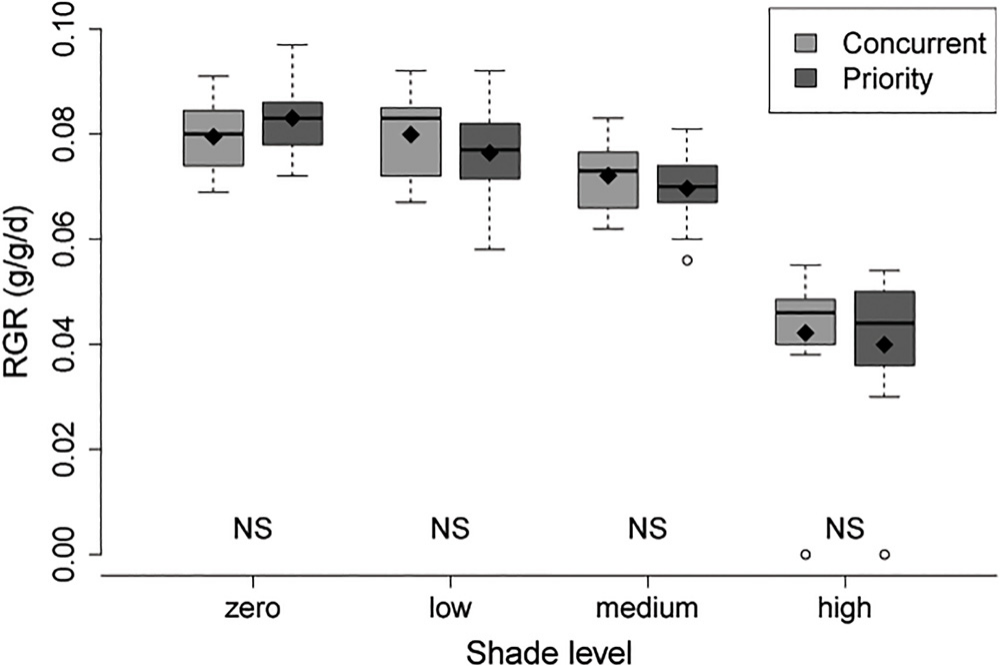
Boxplots comparing *M*. *spicatum* RGR in the concurrent and priority treatments across shade levels. Box edges mark the 1st and 3^rd^ quartile, and the median is shown with a dark line. Whiskers extend to a maximum of 1.5 x interquartile range outward, and values beyond this are indicated by circles. Diamonds show means of each competition treatment*shade level combination. “NS” indicates non-significant differences in pairwise t-tests between competition treatments.

Plot level survival for *M*. *spicatum* was 100% in both competition treatments. Competition treatment did not significantly affect *M*. *spicatum* growth, and RGRs were similar between treatments in each shade level. Shade level did have a significant effect on *M*. *spicatum* RGR, but there was no significant interaction between shade and competition treatments (Table 3B, Fig. 4). Therefore, Tukey means comparisons were performed on the main effect of shade. Mean RGR of *M*. *spicatum* was highest in the zero and low shade treatments, which were not significantly different from each other (p = 0.47). Mean RGR in the medium shade treatment was significantly lower than in the zero (p <0.0001), and low (p = 0.009) shade treatments, and the high shade treatment was significantly lower than the other three (p < 0.0001). Compared to the average of the zero and low shade treatments, mean RGR was 11% lower in medium shade and 49% lower in high shade.

## Discussion

### Light level affects relative performance of *M*. *spicatum* and *E*. *nuttallii*

Competitive suppression of native submersed macrophytes by non-native invasives such as *M*. *spicatum* is widely regarded as problematic [9], but there is little information on how factors such as resource availability or priority effects enhance or reduce these competitive effects. While some experiments have tested macrophyte competition in different nutrient levels (e.g., [35–37]), experiments on the effects of light availability on submersed macrophyte growth are generally conducted on plants grown individually or with conspecifics (e.g., [32,38–40]). My experiments tested the effect of light availability on relative performance of a native and an invasive submersed macrophyte when planted in a competitive environment, which is arguably more relevant to natural systems.

I expected performance of *M*. *spicatum* to decrease relative to that of *E*. *nuttallii* with increasing shade, consistent with the commonly-observed pattern that invasive species have a performance advantage in high resource conditions while natives have an advantage in low resource conditions [3]. However, relative performance of the two species varied with shade level in a different and unexpected way: the two species had similar growth rates in the low and high shade levels, but *M*. *spicatum* had significantly higher growth rates than *E*. *nuttallii* in the zero and medium shade levels (Fig. 2).

This pattern could be attributed to greater competitive abilities of *M*. *spicatum* compared to *E*. *nuttallii* at the zero and medium shade levels and similar competitive abilities in the low and high shade levels. Alternatively (or in part), it could be due to differences in the shape of the growth response to shade for each species independently. If competition was limiting growth, negative correlations between the two species’ growth rates within each shade level might be expected. However, the positive correlations between *E*. *nuttallii* and *M*. *spicatum* growth rates (Fig. 3), suggests both species were responding similarly to conditions in individual plots that may have varied spatially (e.g., moderate differences in soil nutrients in different plots), and that competition was not limiting growth. This lack of competitive suppression may have been due to the abilities of both species to expand laterally within a plot, and implies that competition may not be important between these two species in the early establishment phase, given adequate soil nutrients.

It is likely that the different patterns in growth rate between the two species across shade levels were caused by individual species-specific growth responses to light. *Myriophyllum spicatum* had highest growth rates in the zero and low shade levels, moderately lower growth rates in the medium shade level, and substantially lower growth rates in the high shade level. This pattern is similar to that found by Zefferman [32] when *M*. *spicatum* was planted under the same four shade levels with conspecifics. The similar growth rates in the zero and low shade levels are probably due to light saturation: Su et al. [41] identified the saturation point of *M*. *spicatum* as 1000 μmol photons m^-2^ s^-1^, which is lower than the light levels I measured below water in the zero and low shade treatments at mid-day. *Elodea nuttallii* also had the lowest growth rate in the highest shade level, but in contrast to *M*. *spicatum*, had decreased growth in full light. Although this decrease in the zero shade treatment was not statistically significant, it suggests a photoinhibitory response caused by clear, shallow water and intense mid-summer radiation. Though photoinhibition is rarely documented in freshwater macrophytes, photoinhibitory effects have been found in *E*. *nuttallii* by Hussner et al. [42] and Zefferman [32]. The demonstrated potential for photoinhibition in macrophytes illustrates the importance of conducting experiments in natural light conditions. Even in a glasshouse, UV from the sun may be diminished to an extent that affects macrophyte performance.

*Elodea nuttallii* showed much greater within-treatment variability in growth rates than *M*. *spicatum* (Fig. 2). This was also found by Zefferman [32] and suggests a difference in sensitivity to factors such as water velocity or spatial variation in soil nutrients. Observationally, it appeared that *E*. *nuttallii* grew larger in plots with moderately higher flow, as occurred near the water inputs.

High resources are generally thought to favor invasive over native species (reviewed in Daehler [3]), as invasives often have traits that are associated with high resource acquisition. When the opposite is found, it may be attributed to superior resource conservation traits in the invasive species [5]. My results show that relative performance between natives and invasives across resource levels can have a complex pattern when individual species response curves differ. In this case, *E*. *nuttallii* appears to be sensitive to light at both high and low levels during establishment, while *M*. *spicatum* is sensitive only at low levels. The lack of a linear relationship between light availability and relative performance of these two macrophytes has potentially complicated implications for managers who may wish to use canopy shading to control relative abundance of these species or species with similar growth response curves.

It should be noted that in contrast to many invasive-native species pairs, this particular pair shows an interesting quality: each species is invasive in the other’s home range. In fact, many aquatic plants are invasive outside their home ranges [27]. In this way, using ‘native’ and ‘invasive’ as guild designations for purposes of comparing plant traits may be less relevant in aquatic systems than in terrestrial systems.

### No effect of native priority on *M*. *spicatum*

Priority planting of desirable (often native) species is another method commonly used to reduce invasive weed growth in terrestrial systems (reviewed by Vaughn and Young [20]), but that remains under-explored in freshwater systems. In addition, interactions between resource level and priority effects in any plant community are even more rarely addressed. Priority effects may be particularly important in streams after restoration activities like channel modification, during which existing submersed macrophyte communities may be wiped out. I expected growth rates and survival of *M*. *spicatum* to be reduced where *E*. *nuttallii* was planted five weeks before *M*. *spicatum*, and to show the greatest difference in growth rates in higher light treatments. However, planting *E*. *nuttallii* earlier appeared to have no measurable effect on growth rates of *M*. *spicatum* (Fig. 4) and had no effect on plot level survival, which was 100% in both competition treatments. This result was likely due to the failure of *E*. *nuttallii* to grow as quickly as needed to preempt resources during the initial phase of the experiment, when *E*. *nuttallii* was planted alone in the priority treatment plots. The final biomass of *E*. *nuttallii* was similar for both competition treatments, despite having a five week head start in the priority treatment (Table 2).

The poor performance of *E*. *nuttallii* planted earlier appeared to be caused by insect herbivory. After initial flooding of the channels, I observed a flush of aquatic insect colonization. The number of invertebrate larvae in the channels decreased over time, presumably as insects emerged as adults and predators consumed insect larvae. Much of the *E*. *nuttallii* planted earlier appeared to have herbivory damage, but I did not observe damage to *E*. *nuttallii* planted later. While herbivory by insects on living macrophytes is often thought to be relatively unimportant in freshwater systems, it is not uncommon and has been documented in many cases [43].

This unexpected failure to demonstrate a priority effect illustrates an important consideration in the study of priority effects in general: that relative time is often conflated with real time. In other words, it is difficult to disentangle the effects of an establishment advantage with differences in environmental conditions at the time of plantings, such as differences in temperature, photoperiod, presence and abundance of other species, etc. In addition, there appears to have been a strong year effect (*sensu* Vaughn and Young [20]) on the performance of *E*. *nuttallii*. This experiment was an extension of a study I conducted one year earlier (Zefferman [32]) using the same experimental location at the same time of year, with the same species collected from the same area, yet I observed rapid initial growth of *E*. *nuttallii* in the previous year’s experiment and no obvious herbivory.

The limited number of studies in flowing systems that address the usefulness of native establishment priority as a management technique have yielded mixed results. Native macrophytes can be successfully established in degraded or newly-created stream reaches [32,44], but this process can be difficult and expensive, and new plantings are subject to being washed away [45]. Establishing natives has been shown to limit the spread of invasive macrophytes in restored stream reaches with little weed pressure [21], but not in streams where invasive macrophytes have been weeded but not removed entirely [45]. Furthermore, establishing native submersed macrophytes early may have both inhibitory and facilitative effects in flowing systems. For example, invasion success of *Hydrilla verticillata* was hindered by the presence of established native *Vallisneria americana* in closed mesocosms due to nutrient preemption; however, in the field this inhibitory effect was offset by the constant influx of nutrients and the facilitative effect of established macrophytes catching propagules of the invasive *Hydrilla* [22].

The results of this experiment and others suggest that simply planting native macrophytes is not enough to reduce invasion and spread by invasive submersed macrophyte propagules. To sufficiently preempt space and nutrients, native macrophytes may need considerable time to establish and grow before invasive propagules reach the area, which may or may not be feasible in actual streams. Of course, priority plantings of macrophytes under different physical conditions, native stem densities, and species may have different outcomes than those of my experiment. However, in general, the logistical difficulty of successfully establishing high densities of native macrophytes, the threat of propagule catching, and the influx of nutrients (which may stymie the effects of nutrient preemption) may create exceptional challenges to using priority effects as a management tool in streams compared to terrestrial systems.

## Supporting Information

S1 Dataset Experimental Data. (XLSX)
