# Correction: Experimental Tests of Priority Effects and Light Availability on Relative Performance of *Myriophyllum picatum* and *Elodea nuttallii* Propagules in Artificial Stream Channels

**DOI:** 10.1371/journal.pone.0128049

**Published:** 2015-05-11

**Authors:** The PLOS ONE Staff

Fig 1 is incorrect. The publisher apologizes for the error. Please view the correct version of Fig 1 here.

**Fig 1:**
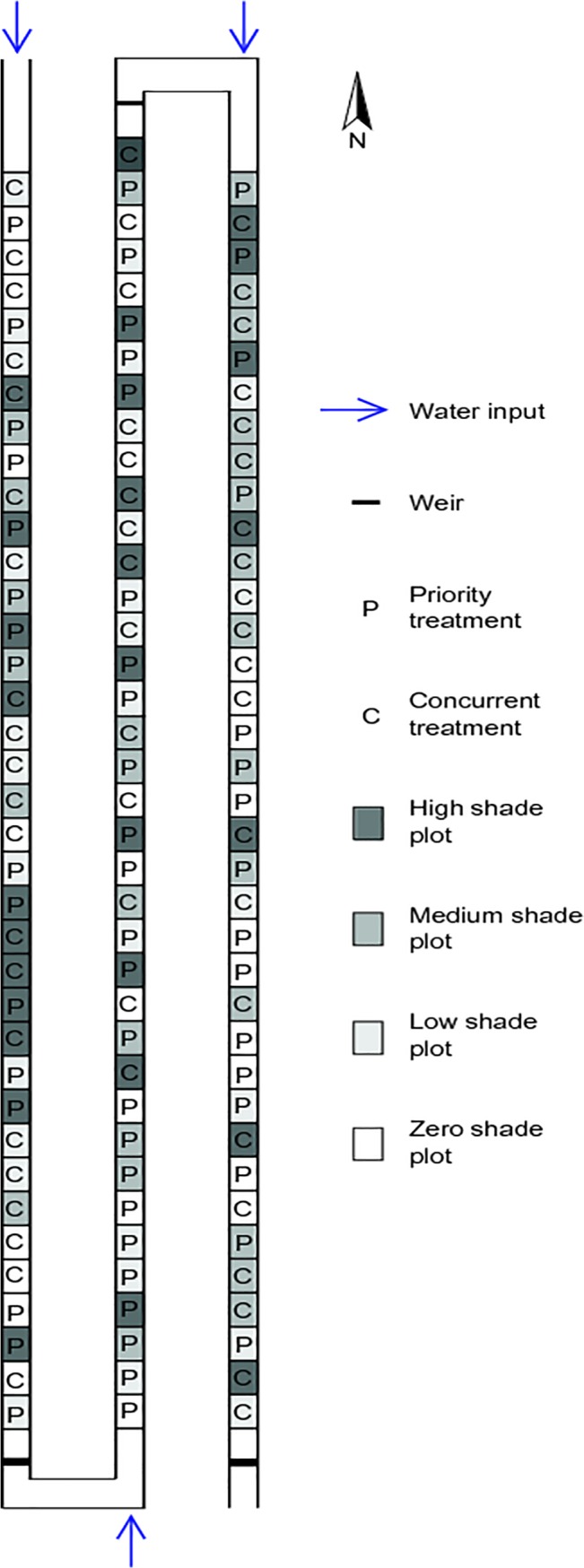
Diagram of experimental layout in artificial channels. Water flowed through the entire channel system with inputs at the upstream end of each of the three channels.
